# Fat Tissue Accretion in Children and Adolescents: Interplay between Food Responsiveness, Gender, and the Home Availability of Snacks

**DOI:** 10.3389/fpsyg.2016.02041

**Published:** 2017-01-04

**Authors:** Annelies De Decker, Sandra Verbeken, Isabelle Sioen, Ellen Moens, Caroline Braet, Stefaan De Henauw

**Affiliations:** ^1^Department of Public Health, Faculty of Medicine and Health Sciences, Ghent UniversityGhent, Belgium; ^2^Department of Developmental, Personality and Social Psychology, Faculty of Psychology and Educational Sciences, Ghent UniversityGhent, Belgium; ^3^Department of Food Safety and Food Quality, Faculty of Bioscience Engineering, Ghent UniversityGhent, Belgium; ^4^Department of Health Sciences, Vesalius, University College GhentGhent, Belgium

**Keywords:** feeding behavior, snacks, home availability, child, adiposity, food responsiveness

## Abstract

The appetitive trait “food responsiveness” is assumed to be a risk factor for adiposity gain primarily in obesogenic environments. So far, the reported results are inconsistent in school-aged children, possibly because these studies did not take into account important moderators such as gender and the food-environment. In order to better inform caregivers, clinicians and the developers of targeted obesity-prevention interventions on the conditions in which food responsiveness precedes adiposity gain, the current study investigated if this relationship is stronger in girls and in children exposed to a higher home availability of energy-dense snacks. Age- and sex-independent Fat and Lean Mass Index z-scores were computed based on air-displacement plethysmography at baseline and after 2 years in a community sample of 129 children (48.8% boys) aged 7.5–14 years at baseline. Parents reported at baseline on children's food responsiveness and the home availability of energy-dense snacks. Food responsiveness was a significant predictor of increases in Fat Mass Index z-scores over 2 years in girls but not boys. The home availability of energy-dense snacks did not significantly moderate the relation of food responsiveness with Fat Mass Index z-score changes. The results suggest that food responsiveness precedes accelerated fat tissue accretion in girls, and may inform targeted obesity-prevention interventions. Further, future research should investigate to which food-environmental parameters children high in food responsiveness mainly respond.

## Introduction

The increase in the supply of cheap, energy-dense foods, coinciding with the rise in the global obesity prevalence, is seen as a major driver of the obesity epidemic (Swinburn et al., 2011). However, individual variability in body size exists also in populations exposed to the same food-environment. This variability is thought to be explained by the interaction between individual and environmental factors (Swinburn et al., 2011; French et al., 2012). Considering the current overweight and obesity prevalence in childhood, its health consequences, and its tracking into adulthood (Singh et al., 2008; Daniels, 2009), understanding which individual factors increase the susceptibility to adiposity gain when exposed to energy-dense foods is essential in order to develop effective obesity-prevention interventions (French et al., 2012).

Schachter's externality theory of obesity stated that food intake in obese people is externally controlled, or in other words, is more determined by external cues related to food compared to internal signals of hunger and satiety (Schachter, 1968, 1971). This responsiveness to food-cues was later on proposed to be an individual factor varying on a continuum in the total population, and is further on called “food responsiveness”(FR) (Wardle et al., 2001). FR can be described as a characteristic that determines the interest and motivation to approach and consume food when food-cues are perceived (French et al., 2012). “Food-cues” refer to external stimuli associated with food, such as pictures, smells, tastes or direct views of food. Hence, FR is assumed to be a risk factor for excess energy intake and adiposity gain primarily in obesogenic environments (Wardle et al., 2001; French et al., 2012). However, before developing interventions that target high FR children and their food-environment, longitudinal studies are needed to confirm this assumption.

In infants, two studies found that FR indeed antecedes accelerated weight gain (van Jaarsveld et al., 2014; Quah et al., 2015). Conversely, results of longitudinal studies in school-aged children, all using the FR-subscale of the Child Eating Behavior Questionnaire (CEBQ) to assess FR (Wardle et al., 2001), were inconsistent: (a) the association between FR and weight gain was non-significant or significant depending on the statistical model used in a community sample of children aged 6 years at baseline and followed over 2 years (this sample had more children with emotional and behavioral problems compared to the community, but was comparable to the community concerning parameters such as parental education level and children's body mass index) (Steinsbekk and Wichstrøm, 2015; Steinsbekk et al., 2016), and (b) the association between FR and weight gain was non-significant after 1 year of follow-up in a community sample of children aged 7–10 years at baseline (Rodenburg et al., 2012). Although it is suggested that FR may only predict accelerated weight gain in infancy, and from then, children high in FR remain on a higher Body Mass Index (BMI) percentile (Rodenburg et al., 2012), the previous studies have also limitations that may explain the inconsistent findings. First, the previous longitudinal studies did not consider an interaction between FR and the food-environment, although an effect of FR on weight gain is mainly assumed in environments plenty of energy-dense foods (Wardle et al., 2001; French et al., 2012). Second, the previous longitudinal studies did also not consider gender differences in the relation between FR and weight gain. There are clear gender differences in the regulation of body composition, which are assumed to be the result of gender differences in biological and socio-psychological processes (Cepeda-Benito et al., 2003; Davy et al., 2006; Shi and Clegg, 2009; Yean et al., 2013). Biological arguments to presume that the relation between FR and weight gain may differ in boys and girls are (a) that the effect of a genetic polymorphism related to higher FR scores, namely the fat and obesity-associated transcript (FTO) gene minor allele on rs9939609 (Velders et al., 2012), on weight and weight gain was only significant among girls in two studies (Jacobsson et al., 2008; Zhang et al., 2014), and (b) that gene vs. food-environment interactions are recently suggested to be more pronounced in females compared to males (van Strien et al., 2015; Silveira et al., 2016). An example of socio-psychological arguments to presume a gender difference is the higher internalization of societal pressure to modify physical appearance in girls compared to boys, leading via higher body dissatisfaction to dietary restraint, which is associated with disinhibited eating episodes (Ruderman, 1986; Stice and Shaw, 2002; Yean et al., 2013).

Knowledge on the interaction of FR with the food-environment and with gender on adiposity gain may help to adequately inform children, caregivers, clinicians, and developers of targeted childhood obesity-prevention interventions on the conditions in which FR precedes adiposity gain. Hence, the goal of this study was to provide, to our knowledge, the first examination of the moderating effect of gender and the food-environment on the relation between FR and adiposity gain in a community sample of children and adolescents aged 7.5–14 years at baseline and 9.5 to 16 at follow-up. Thereby, a stronger positive relation is hypothesized in girls and in children living in a food-environment plenty of energy-dense foods. Since an important part of these children's environment is the home environment, the parameter “home availability of fat- and sugar-rich snacks” (HAS) was included as food-environmental parameter. Children can consume unhealthy foods at home only if these foods are available at home, which is mostly dependent on parental purchases (Campbell et al., 2007). Hence, when confirming an interaction, HAS can be targeted in interventions.

## Methods

### Participants and study design

Subjects were Dutch-speaking Belgian children and adolescents that participated in a population-based study called “Rewarding-FOod ChoicES” (Forces; part of the REWARD-project, www.rewardstudy.be), with study surveys in the spring of 2013 (i.e., baseline) and 2015 (i.e., follow-up). Only children with full data on FR and HAS in 2013 and body composition in 2013 and 2015 were included, which was the case for 129 out of 300 children participating at baseline. These 129 children (in most cases accompanied by at least one parent) attended the survey center at a prefixed appointment both at baseline and follow-up, on which body composition measurements (see below) of the child were conducted. The parental Forces questionnaire of the survey of 2013 was filled in at the survey center or at home; this questionnaire started with socio-demographic questions, followed by the Behavioral Activation System/Behavioral Inhibition System scale, the Behavioral Rating Inventory of Executive Function, the Child Feeding Questionnaire, the Strengths and Difficulties questionnaire, the Environment subscale of the Comprehensive Feeding Practices Questionnaire, the Child Eating Behavior Questionnaire, questions on sleep duration, a 43-item food frequency questionnaire and questions on medical conditions. Of the other 171 participating children at baseline, 103 lacked body composition or FR data at baseline (due to time constraints of children or parents, a part of the children were measured at school instead of at the study center, and consequently, had no BOD POD® measurement), and the other 68 were lost at follow-up.

Participant-recruitment of the Forces study was conducted in two ways: (a) all participants of the control region Aalter of the preceding longitudinal “Identification and prevention of dietary- and lifestyle-induced health effects in children and infants” study (IDEFICS) (Ahrens et al., 2011) were invited via mail, email and, if no response, telephone calls to participate, and (b) children outside of the IDEFICS-cohort of Aalter with an age similar to that of the existing cohort (i.e., 7.5–14 years) were invited at baseline to join the study via an advert on one Flemish television channel and in local newspapers. For the latter recruitment, the only inclusion criterion was age. For the recruitment of the preceding IDEFICS study, the inclusion criteria were age and being a pupil of the 12 schools located in the city Aalter.

The Forces study was conducted according to the guidelines laid down in the Declaration of Helsinki and its later amendments, and approved by the Ethics Committee of Ghent University Hospital. Written informed consent was obtained from all parents and from children older than 12 years. Younger children gave verbal assent.

### Measures

#### FR

The mean score on the Dutch CEBQ-subscale “FR,” designed to measure how responsive a child is to external food-cues, was used. The five items (i.e., “even if my child is full up s/he finds room to eat his/her favorite food”) need to be scored on a five point Likert-scale anchored from “never” (value “1”) to “always” (value “5”) (Wardle et al., 2001; Sleddens et al., 2008), and were completed by parents at baseline. The Cronbach's alpha of the FR subscale in the current study was comparable to the alpha reported in a study on children aged 6–12 years (0.85 vs. 0.89, respectively) (Santos et al., 2011).

#### HAS

HAS was indexed by the mean score of two items reflecting the home availability of fat- and sugar-rich snacks and originating from the subscale “environment” of the “Comprehensive Feeding Practices Questionnaire.” The items, i.e., “I keep a lot of snack food (potato chips, Doritos, cheese puffs) in my house” and “I keep a lot of sweets (candy, ice cream, cake, pies, pastries) in my house,” need to be scored on a five point Likert-scale anchored from “disagree” (value “1”) to “agree” (value “5”) (Melbye et al., 2011). Only these two instead of the four items of the subscale “environment” were used since Melbye et al. demonstrated that the four items load onto two different factors (one reflecting the availability at home of healthy foods and one of unhealthy foods) (Melbye et al., 2011), which was confirmed by a preliminary principal component analysis on the current study data. The mean score of the two items reflecting availability of energy-dense snacks has been positively related to the weekly consumption frequency of fast food (AD, SV, IS, Van Lippevelde W, CB, Eiben G, Pala V, Reish L, De Henauw S, manuscript in review, 2016). The Cronbach's alpha was 0.64 in the current study.

#### Body composition

At least 2 h before the measurement, children had to refrain from physical activity and food. Height was measured barefooted to the nearest 0.1 cm with a SECA® (model 213, SECA Corp., Hamburg, Germany). Body weight was measured with the BOD POD® balance, and body volume with the BOD POD® air-displacement plethysmography device (software version 4.2.4, Life Measurement, Inc., Concord, CA, 2007), both using standardized procedures (McCrory et al., 1995). In accordance to the manufacturer's guidelines, the BOD POD® was calibrated daily and at each measurement, and children wore tight-fitting bathing suits with swimming caps during the measurement. Thoracic gas volume was predicted by the software with a validated child-specific equation (Fields et al., 2004). The body mass index (BMI, kg/m^2^) and body density (kg/L) were calculated, and the latter was converted into body fat percentage using child-specific conversion factors (Wells et al., 2010). Based on body fat percentage and weight, the fat and fat-free masses (kg) were calculated, which were divided by height squared to obtain the fat mass index (FMI, kg/m^2^) and fat-free mass index, also called lean mass index (LMI, kg/m^2^). To compare BMI, FMI and LMI across children, age- and sex-independent z-scores were computed based on Flemish reference curves for BMI (zBMI) (Roelants et al., 2009), and British reference curves for FMI (zFMI) and LMI (zLMI)(Wells et al., 2012) due to absence of Flemish reference curves for these parameters.

#### Parental education level

The highest parental education level of both parents was first categorized according to the International Standard Classification of Education of 1997 (UNESCO, 1997), and, because of low number in some categories, further categorized in two groups: children with no tertiary educated parents (value “0”) and children with minimum one tertiary educated parent (value “1”).

### Data analyses

The two-sided level of significance was set at *p* < 0.05. Analyses were performed using SPSS (version 22.0, SPSS Inc., Chicago, IL, 2013). Normality of the study variables was checked based on visual inspection of histograms and boxplots, and was acceptable for all variables. Unpaired *t*-tests for gender differences were conducted on the study variables. To test drop-out differences, participants at baseline that were included vs. non-included in the current study were compared with unpaired *t*-tests on baseline age, zBMI, and on FR and HAS if available.

To answer the research questions, hierarchic multiple regression models were conducted. In all models, the zFMI change over 2-years follow-up was the dependent variable. Model 1 included all covariates: baseline zFMI, baseline zLMI, centralized age (i.e., age minus 10 years), gender and parental education level (baseline model). Thereby, baseline zLMI was included because it was significantly positively associated with FR in a preliminary analysis, probably because higher FR may also be caused by higher energy needs. In model 2, FR was added to model 1 to evaluate the main effect of FR. To test if the association between FR and adiposity gain is (a) moderated by gender, the interaction term of FR with gender was added to model 2 (model 3a), (b) moderated by HAS, HAS and the interaction term of FR with HAS were added to model 2 (model 3b), and (c) moderated by both gender and HAS, the two-way interactions of FR with gender and HAS with gender and the three-way interaction of FR, HAS and gender were added to model 3b (model 3c).

## Results

**Figure 1:**
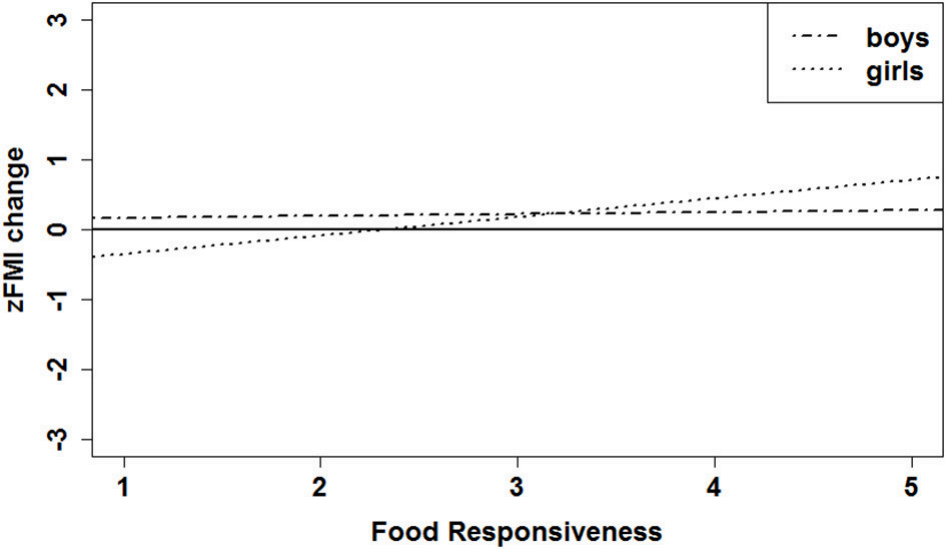
**Estimated effect of FR on zFMI change over 2 years; estimated regression lines are shown for boys and girls with a zFMI baseline = 0, zLMI baseline = 0, age = 10 years, and parental education level = low (i.e., all covariates set at zero)**. zFMI and zLMI, age- and sex-independent z-score of the fat and lean mass index, respectively.

First, the relation between FR and zFMI change was significantly different in boys vs. girls (Table 1, model 3a; significant interaction term between FR and gender). In boys, the relation between FR and zFMI was not significant (Table 1, model 3a; non-significant coefficient of FR). In contrast, a significant positive relation between FR and zFMI change was found in girls: the mean zFMI change of girls over 2 years was 0.27 higher for each unit increase in FR (based on data resulting from a rerun of the analysis of model 3a with a recoded gender variable, i.e., value “0” for girls and value “1” for boys: unstandardized regression coefficient of FR = 0.27, standard error = 0.082, standardized regression coefficient = 0.36, *p* = 0.002). Hence, the results indicated FR as a significant predictor of zFMI increase over 2 years in girls, but not in boys. The estimated regression lines for boys and girls are displayed in Figure 1.

**Table 1:** **Effects of FR, Gender, and HAS on zFMI Change over 2 Years (***n*** = 129)**.

**Dependent variable: zFMI change over 2 years**						
**Model**	**R^2^**	**Independent variables**	**b**	**SE**	**β**	***P***
1	0.20	Constant	0.232	0.132		0.081
		Gender	−**0.228**	0.106	−0.176	0.033
2	0.24	Constant	−0.098	0.185		0.596
		Gender	−**0.262**	0.105	−0.202	0.013
		FR	**0.158**	0.063	0.213	0.014
3a	0.26	Constant	0.140	0.219		0.523
		Gender	−**0.763**	0.271	−0.588	0.006
		FR	0.026	0.091	0.035	0.777
		FR^*^Gender	**0.240**	0.120	0.344	0.048
3b	0.24	Constant	0.100	0.521		0.847
		Gender	−**0.262**	0.106	−0.202	0.015
		FR	0.115	0.246	0.154	0.643
		HAS	−0.080	0.196	−0.096	0.685
		FR^*^HAS	0.018	0.092	0.078	0.847
3c	0.28	Constant	0.471	0.709		0.508
		Gender	−1.380	1.035	−1.064	0.185
		FR	0.043	0.354	0.058	0.903
		HAS	−0.133	0.276	−0.160	0.631
		FR^*^HAS	−0.003	0.135	−0.015	0.980
		FR^*^Gender	0.286	0.486	0.561	0.558
		HAS^*^Gender	0.244	0.389	0.531	0.532
		FR^*^HAS^*^Gender	−0.021	0.182	−0.113	0.909

*zFMI and zLMI, age- and sex-independent z-score of the fat and lean mass index, respectively; b, unstandardized regression coefficient; SE, standard error of b; β, standardized regression coefficient; FR, Food responsiveness; HAS, home availability of fat- and sugar-rich snacks; P < 0.05. Multiple hierarchic linear regression models adjusted for baseline zFMI, zLMI, centralized age (i.e., age minus 10 years), gender (shown in table; value zero for boys, value one for girls) and parental education level. Bold indicates coefficient is significantly different from zero at p < 0.05 level*.

The sex-difference in the relation between FR and zFMI change could not be explained by sex-differences on parameters relevant for the analyses, as baseline age, FR, HAS, zFMI, zLMI and zBMI as well as follow-up zFMI, zLMI and zBMI of participating boys and girls did not significantly differ (Table 2). Further, the results could not be explained by drop-out differences, as no drop-out differences were found on the relevant parameters “zBMI, FR and HAS.” Nevertheless, it was found that the 129 children who were included compared to the 171 children who were excluded from the current study had a lower baseline age, although this probably cannot explain the current result of a sex difference. Further, the study population mainly consisted of children with minimum one tertiary educated parent (79.8%) and of children without overweight or obesity (2.3% had overweight including obesity according to the International Obesity Task Force definition (Cole and Lobstein, 2012).

**Table 2:** **Descriptive statistics of the study population**.

	**Total sample (*n* = 129)**		**Boys (*n* = 63)**		**Girls (*n* = 66)**		***T*-test gender**	
	**Mean**	**sd**	**Mean**	**sd**	**Mean**	**sd**	***t***	***P***
Age at baseline (years)	10.451	1.546	10.522	1.592	10.383	1.509	0.509	0.612
FR (range 1–5)	2.105	0.877	2.029	0.824	2.179	0.925	−0.972	0.333
HAS (range 1–5)	2.601	0.787	2.603	0.799	2.598	0.781	0.034	0.973
zFMI at baseline	−0.563	0.893	−0.514	0.905	−0.609	0.885	0.603	0.548
zFMI at follow-up	−0.351	0.888	−0.313	0.991	−0.456	0.895	1.814	0.072
zLMI at baseline	−0.386	0.942	−0.207	0.821	−0.489	0.934	0.861	0.391
zLMI at follow-up	−0.533	1.020	−0.678	1.085	−0.395	0.941	−1.589	0.114
zBMI at baseline	−0.369	0.792	−0.441	0.770	−0.300	0.812	−1.009	0.315
zBMI at follow-up	−0.316	0.827	−0.427	0.831	−0.209	0.816	−1.505	0.135

*sd, standard deviation of the mean; FR, food responsiveness; HAS, home availability of fat- and sugar-rich snacks; zFMI, age- and sex-independent z-score of the fat mass index based on British reference curves; zLMI, age- and sex-independent z-score of the lean mass index based on British reference curves; zBMI, age- and sex-independent z-score of the body mass index based on Flemish reference curves. P < 0.05. Unpaired t-test for gender differences; degrees of freedom is 127*.

Second, HAS (Table 1, model 3b) nor both HAS and gender (Table 1, model 3c) did significantly moderate the relation between FR and zFMI change.

## Discussion

In the current study, gender was found to moderate the relation between FR and adiposity gain in a community sample of children and young adolescents. However, this relation was not moderated by HAS or by both HAS and gender.

First, a significant positive association between FR and zFMI change over 2 years was found in girls but not boys, independent of common age- and sex-related increases in fat tissue mass and of their baseline zFMI. To our knowledge, only one longitudinal study reported that FR preceded greater BMI gain in school-aged children (Steinsbekk and Wichstrøm, 2015), whereas this association was non-significant in other studies (Rodenburg et al., 2012; Steinsbekk et al., 2016). The inconsistent results of the previous studies may be explained by not considering gender differences, or by using zBMI gain (which may represent zFMI gain but also zLMI gain) as outcome. Conversely, the findings of a gender difference in the current study may also be explained by methodological issues. For example, it is possible that parents have different perspectives on what is adequate responsiveness to food in boys compared to girls. However, food responsiveness scores did not significantly differ between boys and girls in the current study and neither in another study on children with a similar age range, namely 9–12 years (Gonçalves et al., 2012). Additionally, the above mentioned previous studies did use the same questionnaire to assess FR (Rodenburg et al., 2012; Steinsbekk and Wichstrøm, 2015; Steinsbekk et al., 2016). Furthermore, the current results suggesting a gender difference are consistent with findings of a similar gender difference on the association between weight (gain) and the FTO rs9939609 minor allele, (Jacobsson et al., 2008; Zhang et al., 2014) which has been associated with FR (Velders et al., 2012). In sum, FR may precede an augmented adiposity gain over time, and therefore, may contribute to the risk of developing overweight in girls. The suggested augmented fat tissue accretion in girls might be explained by regular energy intake beyond energy needs as FR is per definition a tendency to be highly responsive to external food-cues, and the used FR measure (i.e., subscale “FR” of the CEBQ) has been positively associated with energy intake (Carnell and Wardle, 2007; Hall et al., 2011). However, the found gender difference need to be confirmed by future research.

Explanations of the gender difference found in the current study remain speculative. As mentioned in the introduction, clear gender differences in the regulation of body composition are assumed to be the result of biological as well as psychological processes (Cepeda-Benito et al., 2003; Davy et al., 2006; Shi and Clegg, 2009; Yean et al., 2013) One hypothesis based on biological factors to explain the gender difference may be that, due to adaptive importance for reproduction, the interaction between individual factors and the food-environment is more pronounced in females. This hypothesis was previously suggested by Silveira et al. to explain gender differences in the moderating effect of the environment on the relation between a genetic polymorphism and food consumption (Silveira et al., 2016). Further, socio-psychological factors may also play a role. An example is the higher internalization of societal pressure to modify physical appearance, the higher body dissatisfaction and the higher drive for thinness in girls compared to boys (Yean et al., 2013). These factors compose a risk to develop disordered eating behavior via increases in weight-control intentions: dietary restraint often precedes disinhibited eating episodes at instances when self-control ability decreases (Ruderman, 1986; Stice and Shaw, 2002).

If the relation between FR and adiposity gain in girls is confirmed, obesity-prevention efforts may benefit from targeting high FR girls with tailored interventions and psycho-education. In school-aged children, interventions tailored to high FR girls may consist of recent innovative strategies aiming to reduce overeating in response to external food cues based on Schachter's externality theory (Schachter, 1971); (a) strategies to increase the girls' appetitive awareness by developing a greater sensitivity to hunger and satiety (Boutelle et al., 2014a); (b) strategies to strengthen self-control skills to inhibit automatic approach responses to external food cues (Appelhans et al., 2011; Verbeken et al., 2013); (c) strategies to extinguish the learned response of eating in response to external food cues (Boutelle et al., 2014a; Boutelle and Bouton, 2015); (d) strategies to train attention away from food to non-food cues (Boutelle et al., 2014b, 2016), and (e) strategies in order to adapt the food-environment, e.g., reduce portion sizes (Mooreville et al., 2015). Hence, the previous strategies aim to learn high FR children and their parents to adequately handle the children's high responsiveness to external food cues. Certainly in young adolescents that gain autonomy over their food intake, it is important that the high FR adolescents themselves learn all of the above mentioned strategies (Todd et al., 2015). As such, they learn how to rely on internal hunger and satiety signals for the self-regulation of food intake (also called “intuitive eating,” an eating style that is inversely related to BMI) and how to prevent that external cues influence food intake (Herbert et al., 2013). Furthermore, recent research in infants and toddlers suggest that it may even be possible to prevent the development of excessive FR levels by means of early psycho-education to parents, which (a) promotes the use of early feeding practices such as mere exposure to healthy food, limiting exposure to unhealthy food and feeding that recognizes and responds appropriately to cues of hunger and satiety (Daniels et al., 2014; Magarey et al., 2016), and (b) discourage the parenting strategies “using food as reward for children's behavior” and “using food to influence a child's emotions” (Carnell et al., 2014; Mallan et al., 2016).

Secondly, this study investigated the assumption that FR precedes adiposity mainly in environments plenty of energy-dense foods, using HAS as food-environmental parameter (Wardle et al., 2001; French et al., 2012). However, no significant moderating effect of HAS or of HAS and gender was found. It is difficult to explain these results, since the current study is to our knowledge the first to investigate this with the distal outcome “adiposity gain.” However, the assumption has already been experimentally investigated with the more proximal outcome “energy-intake” (Mooreville et al., 2015), whereby high FR children were found to be susceptible to excess energy intake when portion sizes increase. Possibly, high FR children may be more vulnerable to the amounts of food being served or to still other parameters of the home food-environment (e.g., parameters that also include drinks or foods eaten at mealtime) than to the mere availability of energy-dense snacks at home. Further, only one element of the enormous diversity of food-environmental elements that currently influence eating behaviors of children and adolescents was assessed in the present study. Although the importance of the home food availability for children's and adolescents' food consumption has been shown in previous research (Campbell et al., 2007; Vereecken et al., 2010; Cutler et al., 2011; Johnson et al., 2011; Pearson et al., 2014), the influence of the obesogenic environment outside home on adiposity gain in high FR primary school children and adolescents might be preponderant over the influence of the home food-environment (Chopra et al., 2002). Children may be exposed to energy-dense foods when spending time with their peers, at school, on the way to and from school, at sport clubs, at the homes of other family members, etc. For example, the availability of unhealthy snacks and drinks at school has already been reported to moderate the association between peer group and individual consumption in adolescents (Wouters et al., 2010). At last, the validity of the HAS measure could be questioned since it is just a two-item measure and since HAS was reported by the parents, which may have a tendency to trivialize an unhealthy food-environment created by themselves. Nonetheless, HAS has already been related to the weekly consumption frequency of fast food (De Decker A, Verbeken S, Sioen I, Van Lippevelde W, Braet C, Eiben G, Pala V, Reish L, De Henauw S, manuscript in review, 2016). Anyhow, it is of interest to use a more comprehensive measure of the food-environment in future studies, including diverse food-environmental aspects in multiple settings (e.g., school, school neighborhood, residential neighborhood, etc.).

The current study was to our knowledge the first longitudinal study on the moderating effect of gender and HAS in the relation between the appetitive trait FR and adiposity gain in primary school children and young adolescents. Strengths of this study include the use of zFMI-scores based on air-displacement plethysmography instead of zBMI-scores, the longitudinal design, the community sample of children, and the use of the short FR-subscale which is easily and practically applicable for prevention purposes. Other limitations of the current study, besides the limitations mentioned in the previous paragraph, include the use of British FMI and LMI reference data due to absence of Flemish reference data. Nevertheless, only minor differences between neighboring British and Flemish populations were seen (standard deviation of zFMI and zLMI on all time points close to one in both sexes). Another limitation is the lack of information on puberty, since the parameter zFMI is adjusted for age and sex, but not puberty. Comparable with the average zBMI based on Flemish reference scores, the average zFMI and zLMI were slightly negative, probably due to an overrepresentation of children from highly educated parents, and therefore, healthier body composition (Lamerz et al., 2005). Furthermore, parental scoring of FR as well as HAS may be influenced by social desirability, the study did not take into account other parental feeding practices, and a time span of 2 years may be too short to find effects of HAS on a distal measure such as fat tissue.

In conclusion, although it remains speculative why the appetitive trait FR might be a risk factor for higher fat tissue accretion in girls but not boys, obesity-prevention efforts may benefit from targeting high FR girls with tailored interventions. Future research is needed (a) to further investigate to which food-environmental parameters children high in FR mainly respond, (b) to evaluate if the above mentioned intervention strategies are effective in preventing excess adiposity gain in high FR girls, (c) to confirm the gender-specific effect, best in larger cohorts, and if so, (d) to investigate the mechanisms underlying the gender difference.

## Author contributions

All authors made substantial contributions to the conception and design of the study and interpretation of data, and approved the final paper as submitted. AD carried out the analyses, made the figure and drafted the paper, the other authors critically revised the paper. AD, I. Sioen, and SV organized the data collection.

## Funding

This work was supported by Flanders Innovation and Entrepreneurship (grant number SBO-120054), and the Research Foundation—Flanders (I. Sioen, grant number 1.2.683.14.N.00).

### Conflict of interest statement

The authors declare that the research was conducted in the absence of any commercial or financial relationships that could be construed as a potential conflict of interest.

## Abbreviations

CEBQ: Child Eating Behavior Questionnaire
FR: Food responsiveness
FTO: fat and obesity-associated transcript gene
HAS: home availability of energy-dense snacks
zBMI: age- and sex-independent z-score of the Body Mass Index
zFMI: age- and sex-independent z-score of the Fat Mass Index
zLMI: age- and sex-independent z-score of the Lean Mass Index.
